# Latitudinal Variations in Seasonal Activity of Influenza and Respiratory Syncytial Virus (RSV): A Global Comparative Review

**DOI:** 10.1371/journal.pone.0054445

**Published:** 2013-02-14

**Authors:** Kimberly Bloom-Feshbach, Wladimir J. Alonso, Vivek Charu, James Tamerius, Lone Simonsen, Mark A. Miller, Cécile Viboud

**Affiliations:** 1 Fogarty International Center, National Institutes of Health, Bethesda, Maryland, United States of America; 2 Mount Sinai School of Medicine, New York, New York, United States of America; 3 School of Medicine, Johns Hopkins University, Baltimore, Maryland, United States of America; 4 The Department of Environmental Health Sciences, Columbia University, New York, New York, United States of America; 5 Department of Global Health, George Washington University School of Public Health and Health Services, Washington, D. C., United States of America; University of Hong Kong, Hong Kong

## Abstract

**Background:**

There is limited information on influenza and respiratory syncytial virus (RSV) seasonal patterns in tropical areas, although there is renewed interest in understanding the seasonal drivers of respiratory viruses.

**Methods:**

We review geographic variations in seasonality of laboratory-confirmed influenza and RSV epidemics in 137 global locations based on literature review and electronic sources. We assessed peak timing and epidemic duration and explored their association with geography and study settings. We fitted time series model to weekly national data available from the WHO influenza surveillance system (FluNet) to further characterize seasonal parameters.

**Results:**

Influenza and RSV activity consistently peaked during winter months in temperate locales, while there was greater diversity in the tropics. Several temperate locations experienced semi-annual influenza activity with peaks occurring in winter and summer. Semi-annual activity was relatively common in tropical areas of Southeast Asia for both viruses. Biennial cycles of RSV activity were identified in Northern Europe. Both viruses exhibited weak latitudinal gradients in the timing of epidemics by hemisphere, with peak timing occurring later in the calendar year with increasing latitude (P<0.03). Time series model applied to influenza data from 85 countries confirmed the presence of latitudinal gradients in timing, duration, seasonal amplitude, and between-year variability of epidemics. Overall, 80% of tropical locations experienced distinct RSV seasons lasting 6 months or less, while the percentage was 50% for influenza.

**Conclusion:**

Our review combining literature and electronic data sources suggests that a large fraction of tropical locations experience focused seasons of respiratory virus activity in individual years. Information on seasonal patterns remains limited in large undersampled regions, included Africa and Central America. Future studies should attempt to link the observed latitudinal gradients in seasonality of viral epidemics with climatic and population factors, and explore regional differences in disease transmission dynamics and attack rates.

## Introduction

For reasons that are poorly understood, influenza and respiratory syncytial virus (RSV) exhibit distinct winter seasonality in temperate latitudes, while patterns of activity are more diverse in tropical locations [1]–[6]. A better understanding of influenza seasonality is useful to inform the timing and composition of vaccine recommendations and monitor the emergence of new virus variants [7]–[8]. Influenza vaccines have become increasingly available in middle-income countries, but the complex seasonal patterns of virus activity complicate the design of effective vaccination campaigns. In Northern Brazil, for instance, annual vaccination campaigns are timed according to the Southern Hemisphere winter season, which is too late in respect to the local influenza season [8]–[9]. Although no vaccine is currently available to prevent RSV infection, monthly administration of palivizumab, a monoclonal antibody used for prophylaxis in high-risk infants, needs to be timed according to local circulation patterns of the virus [10]. Despite renewed interest in elucidating the mechanisms of seasonality of infectious diseases [1], [3]–[4], [6], [11], the seasonal patterns of influenza and RSV have not been systematically reviewed on a global scale in recent years. In parallel, surveillance efforts for respiratory viruses have been greatly strengthened in the last decade, justifying new analyses of recently collected data.

Several hypotheses have been posited to explain the seasonality of respiratory viruses, although no single factor is sufficient to explain the diversity of seasonal patterns globally [1], [3]–[6], [11]–[12]. Possible mechanisms include seasonal changes in virus survival and transmissibility, host physiology and social behavior, and their interactions [4]. Host susceptibility to infection may increase in winter due to vitamin D deficiency [12], dehydration of mucus in the nose and respiratory tract, or changes in immune functions due to melatonin cycles [1]. Other hypotheses point to changes in social behavior during winter months, such as increased contact due to holiday gatherings and more time spent indoors avoiding the cold. In addition, virus survival may increase in wintertime conditions [4]. Experimental and epidemiological data have suggested that low absolute humidity may increase influenza virus survival and transmission [13]–[15] and affect the timing of onset of seasonal and pandemic outbreaks in the US [16]–[17].

Overall, a global picture of influenza and RSV seasonal patterns is lacking and would be useful to evaluate some of the postulated mechanisms driving disease seasonality, and guide public health strategies globally. Here, we conduct an extensive review of the literature and electronic surveillance websites to identify laboratory-confirmed data on influenza and RSV activity, characterize seasonal patterns globally, and study their association with geographical factors.

## Materials and Methods

Our databases include laboratory-confirmed information on influenza and RSV activity culled from the literature and electronic surveillance websites, which were available at various temporal and spatial resolutions, as detailed below.

### 1. Influenza Literature Search

#### 1.1 Search strategy

A literature search was conducted in Pubmed between May 2009 and February 2010 for influenza and RSV articles published between 1990 and 2009. Keywords included ‘respiratory virus’, OR ‘influenza,’ OR ‘respiratory syncytial virus,’ OR ‘RSV,’ in combination with ‘laboratory’ OR ‘surveillance’. We also used ‘influenza’ OR ‘respiratory syncytial virus’ in combination with geographical locators, including ‘tropics,’ ‘tropical,’ ‘temperate,’ each continent, and regions (i.e. ‘Latin America’ or ‘Middle East’). References cited in retrieved articles were also examined for possible inclusion in this review. We restricted the search to papers in English.

#### 1.2 Inclusion/Exclusion criteria

We included all hospital and community-based studies using one or more laboratory tests to detect influenza, RSV, or both. Studies had to be conducted for one consecutive year or more, and report weekly or monthly counts or percent of virus positive in a table or graphic format. We excluded studies focusing on the 2009 A/H1N1 pandemic to limit the analysis to influenza patterns in inter-pandemic periods. We also excluded studies reporting less than 24 influenza virus (resp. RSV) positive specimens in the year, so that a minimum average of 2 virus-positive specimens per month was required for inclusion in this review.

#### 1.3 Information retrieved

For each study, we recorded the following variables: study location (city, country), years and duration, setting (community, hospital, emergency ward, outpatient clinics), number of individuals and/or respiratory samples tested, number of influenza and/or RSV positive specimens, number of respiratory virus positive specimens, participant age (range, mean, median, or proportion of individuals younger than one year old). In order to characterize influenza and RSV seasonality, monthly counts of viral infections were compiled from tables or digitized from graphs.

### 2. Electronic Sources of Publicly Available Influenza Surveillance Data

Additional publicly available data on weekly or monthly influenza virus isolations were obtained from queries of international and national influenza surveillance systems, including the Australia Department of Health’s National Influenza Surveillance Scheme [18], the European Influenza Surveillance Scheme [19], Japan’s Infectious Disease Surveillance Center [20], the World Health Organization FluNet [21] and the US influenza surveillance system maintained by the Centers for Disease Control and Prevention [22]. We were not aware of publicly available on-line data sources for RSV but we included information from an excellent review of RSV seasonality patterns globally published in 1998 [5].

### 3. Geographical and Socio-economic Information

Latitude and longitude coordinates for study sites were obtained from Falling Rain Genomics, Inc. [23] For multicenter studies that did not break down data by study site, we used the mean coordinates of the study sites. The tropics were defined as locations equatorward of 23°27′ N/S. National economic status was quantified according to the World Bank List of Economies (as of 2009), which assigns countries income ratings of ‘low,’ ‘lower middle,’ ‘upper middle,’ and ‘high.’ [24].

### 4. Overview of Analytical Approach

Given the diversity of data available from electronic surveillance websites and literature data sources, we conducted two sets of seasonality analyses. The first set of analyses was based on simple measures of peak timing and duration that could be applied to relatively sparse data from many different locations, and was appropriate for highly spatially-resolved data available from the literature or from sub-national surveillance websites. In the second set of analyses, we applied more refined time series models to weekly country-specific surveillance data, making use of the systematic influenza data collection system available through the WHO FluNet website [21]. Taken together, these two sets of seasonality analyses provide good balance between spatial and temporal resolutions.

### 5. First set of Analyses Applied to Sub-national Data

#### 5.1. Estimation of peak timing and epidemic duration

The seasonal parameters of interest were estimates of peak month and duration of influenza and/or RSV epidemic activity. Peak month was defined as the month with the maximum number of viruses isolated within the year. If the study covered multiple years, the average peak month was estimated. Epidemic duration was defined as the number of months when viral activity exceeded an epidemic threshold, defined as 5% of the annual number of influenza or RSV viruses, because it appeared to provide a reliable definition of epidemic period. We also used a 10% cut-off to test the sensitivity of our results to the choice of threshold level. For studies reporting weekly data, the month corresponding to the week of peak activity was recorded and the calculation of epidemic duration was modified to be commensurate with the durations calculated form the monthly data (weekly epidemic threshold set at 1.2%).

Some locations were characterized by two distinct epidemic periods within the year. We identified locations experiencing semi-annual activity, based on whether two peaks in viral isolates occurred at least 4 months apart within the same calendar year, and viral activity dropped below the 5% epidemic threshold in-between the two peaks. In most cases of semi-annual activity, we could define a “major” peak (most intense peak, highly consistent between years) and a “minor” peak (less intense and/or less consistent). If two influenza subtypes (eg, A/H3N2 and B) caused two distinct peaks in a single winter season, as can be the case in Northern America and Europe, these peaks could theoretically be counted as “semi-annual activity”. However the 2 peaks would have to be separated by ≥4 months and include one crossing below epidemic threshold. In fact, by this criterion, none of the high-latitude locations in our dataset were classified as having semi-annual peaks in any of the study years.

Of note, no location had constant respiratory virus activity throughout the year when individual years were considered ( = same no. of virus positive specimens in all months of the year), and a peak could always be defined.

#### 5.2. Statistical analyses and maps of peak timing and epidemic duration

Univariate and multivariate analyses using non-parametric Wilcoxon tests and linear regression were used to assess the impact of geography, sample size, and study design, on peak month and epidemic duration. Sensitivity analyses were conducted using regression, weighted by the no. of samples tested. For the purpose of analyzing and mapping peak timing, we recoded peak month using a running index from 1–12, representing the respiratory season running from July-June in the Northern Hemisphere and January-December in the Southern Hemisphere.

Maps of peak activity and epidemic duration were plotted by averaging out independent observations for the same location, separately for influenza and RSV. If the information about periods of virus circulation did not overlap between studies reporting on the same site, or if a ‘semi-annual peak’ of activity had been defined as explained above, a secondary peak was displayed.

### 6. Second Set of Analyses Applied to Weekly National Time Series from FluNet

We conducted separate time series analyses of national surveillance data available from FluNet, as these were systematically collected on a weekly basis in many countries and allowed for more refined estimation of seasonal parameters. The average seasonal signature of influenza in different locations was obtained by fitting the following regression models [25]: *y_t_* = *a*+b*cos (2Pi*t/52.17)+ c*sin (2Pi*t/52.17)+ d*cos (4Pi*t/52.17)+e*cos (4Pi*t/52.17), where *y_t_* is the weekly standardized number of viruses in week *t,* and *a, b, c, d, e* are parameters to be estimated from the data *(b* and *c* indicate the strength of the annual cycle, while *d* and *e* correspond to the semi-annual cycle). Since information on the weekly no. of specimens tested was missing for many years and countries, we standardized the weekly no. of viruses by the cumulative number of viruses reported during the respiratory season (Jul-Jun in the Northern Hemisphere, Jan-Dec in the Southern Hemisphere). To estimate average peak timing and year-to-year fluctuations in timing, we estimated the “center of gravity” separately for each country and season [26], which is obtained by weighting the number of viruses every week by the week number.

Our main analysis was limited to countries reporting ≥30 viruses annually for ≥3 respiratory seasons, after exclusion of the A/H1N1 pandemic period (Jan-Dec 2009 for the Southern Hemisphere; July 2009–June 2010 for the Northern Hemisphere). A period of 3 seasons was considered the minimum to fit time series models. We also provide a sensitivity analysis based on countries reporting more than 100 positive viruses for ≥3 respiratory seasons.

## Results

Our results are organized as follows: first, we present descriptive statistics on the number of study sites identified through our literature search and queries of electronic surveillance websites (Section 1). Second, we present the results of seasonality analyses focused on highly resolved spatial data but using coarse measures of peak timing and duration (Sections 2–4). In the last section (Section 5), we present the results of time series models applied to weekly influenza surveillance data available from FluNet.

### 1. Literature and On-line Search

**Figure 1:**
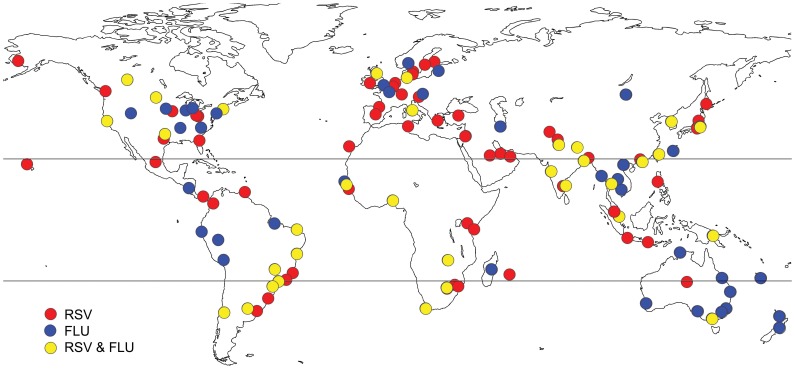
Map of 137 studied locations, for which information was retrieved on the seasonality of influenza or RSV viral activity (blue dot = influenza study, red dot = RSV study, yellow dot = both influenza and RSV information were available from the same study).

The literature search retrieved 164 relevant articles and electronic data sources providing information on influenza and/or RSV seasonality patterns and compatible with our inclusion criteria (See Figure S1 for a flow diagram; Table S1 for a complete list of references, and Figure 1 for a map of studied locations).

Table 1 summarizes viral surveillance information retrieved on a sub national scale. Overall, we identified peak timing in 77 distinct locations for influenza and 96 locations for RSV, covering a latitudinal range of −41°S to 61°N, with one-third of the studies sites in the tropics. Twenty-eight studies provided information on both influenza and RSV seasonal activity in the same location.

**Table 1:** Summary of information retrieved on influenza and respiratory syncytial virus (RSV) viral activity by literature search and queries of electronic surveillance websites providing sub-national data.

Virus	No. articles andelectronic sources	No. countriescovered	No. studysites	Latitudinal range	No. (%) sites inthe tropics	No. (%) providinginformation onepidemic duration
Influenza	85	40	77	−41°S (Wellington, New Zealand)to 60°N (Oslo, Norway)	30 (39%)	47 (61%)
RSV	106	52	96	−38°S (Melbourne, Australia) to61°N (Yukon Delta, Alaska, US)	31 (32%)	63 (59%)
Influenza and RSV	28	18	25	−38°S (Melbourne, Australia) to55.9°N (Edinburgh Scotland)	7 (25%)	16 (60%)

All studies met inclusion criteria (methods) and allowed estimation of month of peak activity for ≥1 year; a subset allowed estimation of epidemic duration.

Based on the WHO global surveillance website FluNet, we identified 85 countries reporting sufficient influenza information amenable to time series model fitting, including 32 (sub)tropical countries (38%).

### 2. Influenza Seasonal Patterns in Sub-national Data

#### 2.1. Influenza Peak Timing

**Figure 2:**
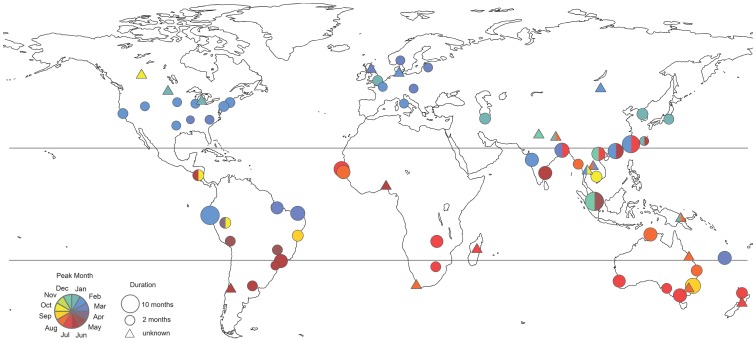
Global map of influenza peak timing and epidemic duration (n = 77 locations). Colors illustrate timing of peak influenza activity, based on the bottom left key, while size of the circles is proportional to epidemic duration. For independent observations for the same location, an average was taken. For studies that did not provide enough information to estimate duration, a triangle is shown. Circles filled out with more than one color represent locations experiencing semi-annual peaks of virus activity (methods).

**Figure 3:**
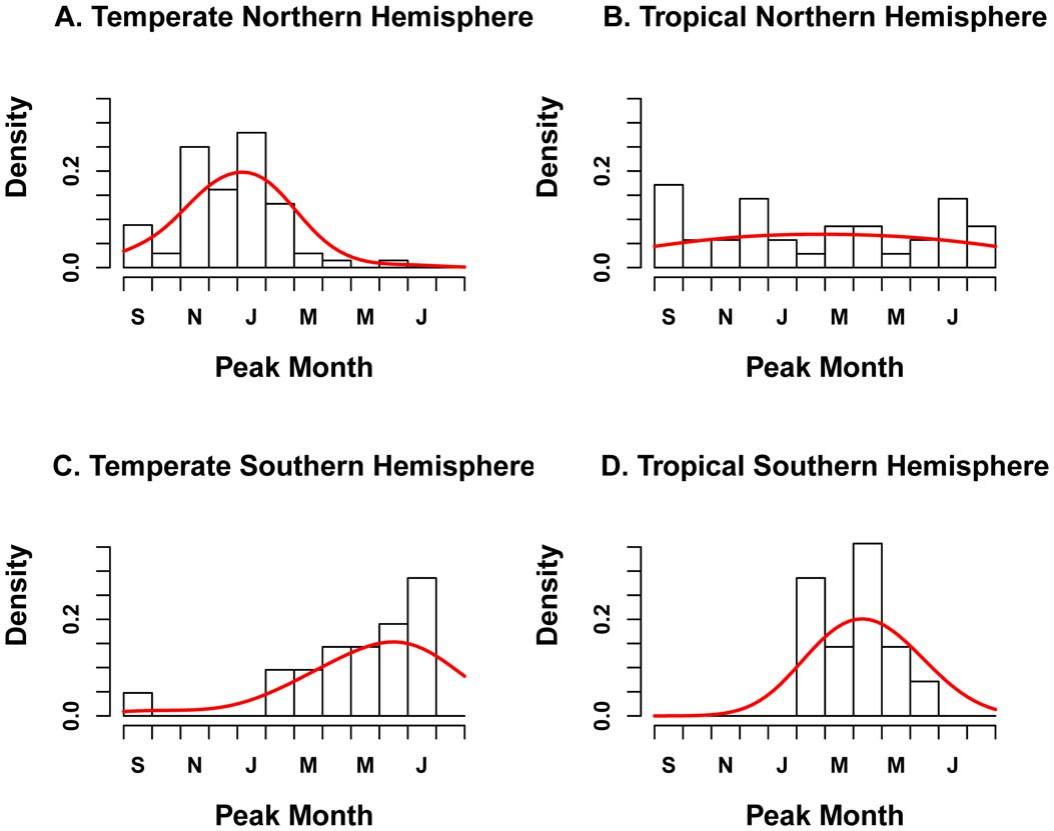
Distribution of influenza peak month by geographic zone (n = 77 locations). The black histogram represents observations while the red curve illustrates the fit of a Gaussian density kernel.

A global map of influenza peak timing and duration is provided in Figure 2. Influenza peaks were highly concentrated in winter months in temperate locations of the Northern Hemisphere with a mode in February (n = 46 distinct locations, Table 2 and Figure 3A). There were 7 outliers in temperate areas of the Northern Hemisphere with peak timing outside of the typical December-March winter months. Five of these studies were set in Asia, one in Saudi Arabia, and one in Canada [27]–[35]. Specifically, October-November influenza peaks were reported in Edmonton, Canada (53.5° N) New Delhi, India (28.6° N) and Riyadh, Saudi Arabia (24.6° N) [27]–[29]. April peaks were reported in Seoul, Korea, in several study years [30]. Multi-year studies in Bhaktapur, Nepal (27.7° N), Okinawa, Japan (26.3° N), and Taipei, Taiwan (26.3° N) revealed patterns of semi-annual influenza activity, with peaks occurring in January-February and June-August of each year [31]–[35]. These summer influenza peaks reported in temperate East and South East Asian locations were remarkably out-of-phase with respect to the typical temperate Northern Hemisphere influenza season.

**Table 2:** Influenza and respiratory syncytial virus (RSV) peak timing and epidemic duration by geographic zone.

Region	Influenza				RSV			
	Peak timing		Epidemic duration*		Peak timing		Epidemic duration*	
	Mode (no.studies)	Median (IQR)	Mean in mo.(no. studies)	Median inmo. (IQR)	Mode (no.studies)	Median (IQR)	Mean in mo.(no. studies)	Median inmo. (IQR)
NH temperate	Feb (n = 51)	Feb (Jan–late-Feb)	4.0 (n = 27)	3 (3–4)	Feb (n = 68)	Jan (Dec–Feb)	4.8 (n = 37)	4 (4–6)
NH tropics	Jul (n = 49)	Jun (Feb–Jul)	6.2 (n = 17)	6 (5–7)	Jan (n = 35)	Mar (Dec–Jul)	4.9 (n = 16)	5 (3–5.5)
SH tropics	Aug (n = 23)	Apr (mid-Feb–Jul)	5.7 (n = 13)	5 (4–7)	Apr (n = 14)	mid-Apr (Mar–May)	5.0 (n = 10)	5 (4–5)
SH temperate	Jul (n = 21)	Jul (Jun–Jul)	5.5 (n = 13)	5 (4–7)	Jul (n = 21)	Jun (May–mid-Jul)	4.5 (n = 14)	4 (4–5)

IQR: Interquartile range; SH: Southern Hemisphere; NH: Northern Hemisphere.

This table provides information on the number of distinct studies included in the review, while information on the number of distinct locations is provided in the text.

* Based on a 5% threshold; see methods for details.

In tropical zones of the Northern and Southern Hemispheres (n = 52), influenza peak timing was more diverse, with several modes (Table 2 and Figure 3B–D). There was no difference in the distribution of peak months in tropical locations of the Southern and Northern Hemispheres (Wilcoxon Test, P = 0.96). The observed variability in peak timing in the tropics was in part due to semi-annual influenza activity, identified predominantly across East and South East Asia, including in Manila, Philippines; Singapore; Nakhon Phanom and Sa Keao, Thailand; Hanoi, Vietnam; and Hong Kong, China [36]–[41]. In these locations, the major peak was reported in winter, and the minor peak occurred in summer.

In temperate locations of the Southern Hemisphere (n = 20), influenza epidemics were concentrated in winter months with a mode in July and inter-quartile range June to mid-July (Table 2 and Figure 3C). There was no report of influenza peaks occurring outside of June-September.

Influenza peak timing was weakly associated with latitude in the Northern Hemispheres (Table 3, P<0.02), with peak timing occurring later in the year with increasing absolute latitude in unweighted and weighted regressions (Figure S2). A similarly pattern was evidenced in the Southern Hemisphere, although the association was only significant in weighted regression (Figure S2). There was no association between influenza peak timing, sample size, study settings or socio-economic factors.

**Table 3:** Association between absolute latitude, peak timing, and duration of influenza and respiratory syncytial virus (RSV) epidemics, by geographic zone.

Epidemic duration ∼ Latitude*						
	Influenza			RSV		
Region	No. studies	Coefficient for latitude (R^2^)	P-value	No. studies	Coefficient for latitude (R^2^)	P-value
All regions	70	−0.07 (R^2^ = 0.18)	**P = 0.0003**	77	−0.02 (R^2^ = 0.03)	P = 0.11
NH	44	−0.09 (R^2^ = 0.35)	**P<0.0001**	53	−0.02 (R^2^ = 0.03)	P = 0.21
SH	26	0.0002 (R^2^<0.001)	P = 0.99	24	−0.04 (R^2^ = 0.102)	P = 0.13
**Peak month ∼ Latitude** *						
	**Influenza**			**RSV**		
**Region**	**No. studies**	**Coefficient for latitude (R^2^)**	**P-value**	**No. studies**	**Coefficient for latitude (R^2^)**	**P-value**
NH	99	0.11 (R^2^ = 0.22)	**P<0.0001**	102	0.05 (R^2^ = 0.09)	**P = 0.001**
SH	44	0.04 (R^2^ = 0.05)	P = 0.16	35	0.08 (R^2^ = 0.24)	**P = 0.003**

Statistically significant P-values appear in bold.

SH: Southern Hemisphere; NH: Northern Hemisphere.

Duration estimates based on a 5% threshold; see methods for details.

This table provides information on the number of distinct studies included in the review, while information on the number of distinct locations is provided in the text.

* Statistics based on linear regression of duration or peak month against latitude.

#### 2.2. Influenza Epidemic Duration

Median epidemic duration for influenza was 5 months, using a 5% epidemic threshold, with an interquartile range of 4–6.5 months. Median epidemic duration was longer in the tropics (6 months) than in temperate areas (4 months; Wilcoxon, P<0.0001). Absolute latitude was a modest predictor of epidemic duration in the Northern Hemisphere, but there was no relationship in the Southern Hemisphere nor in regression weighted by no. of sample tested (Table 3 and Figure S3). Epidemic duration did not vary with study setting (P = 0.5), number of respiratory specimens tested (less or greater than 1000, P = 0.8), number of influenza viruses identified (less or greater than 150, P = 0.96), or study duration (less or greater than 2 years of data, P = 0.06).

A sensitivity analysis using a more stringent 10% threshold for epidemic activity produced substantially shorter estimates of epidemic duration (median = 3 months, interquartile range = 2–4 months) but produced similarly weak associations with latitude (Figure S3). Further sensitivity analyses limited to a subset of influenza studies conducted for over 2 years are provided in Text S1 and confirmed our main findings.

### 3. RSV Seasonal Patterns in Sub-national Data

#### 3.1. RSV Peak Timing

**Figure 4:**
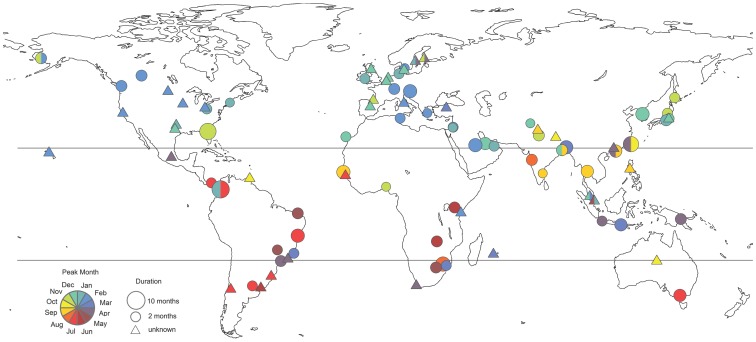
Global map of RSV peak timing and epidemic duration (n = 96 locations). Colors illustrate timing of peak RSV activity, based on the bottom left key, while size of the circles is proportional to epidemic duration. Independent observations for the same location were averaged out. For studies that did not provide enough information to estimate duration, a triangle is shown. Circles filled out with more than one color represent locations experiencing semi-annual peaks of virus activity (methods).

**Figure 5:**
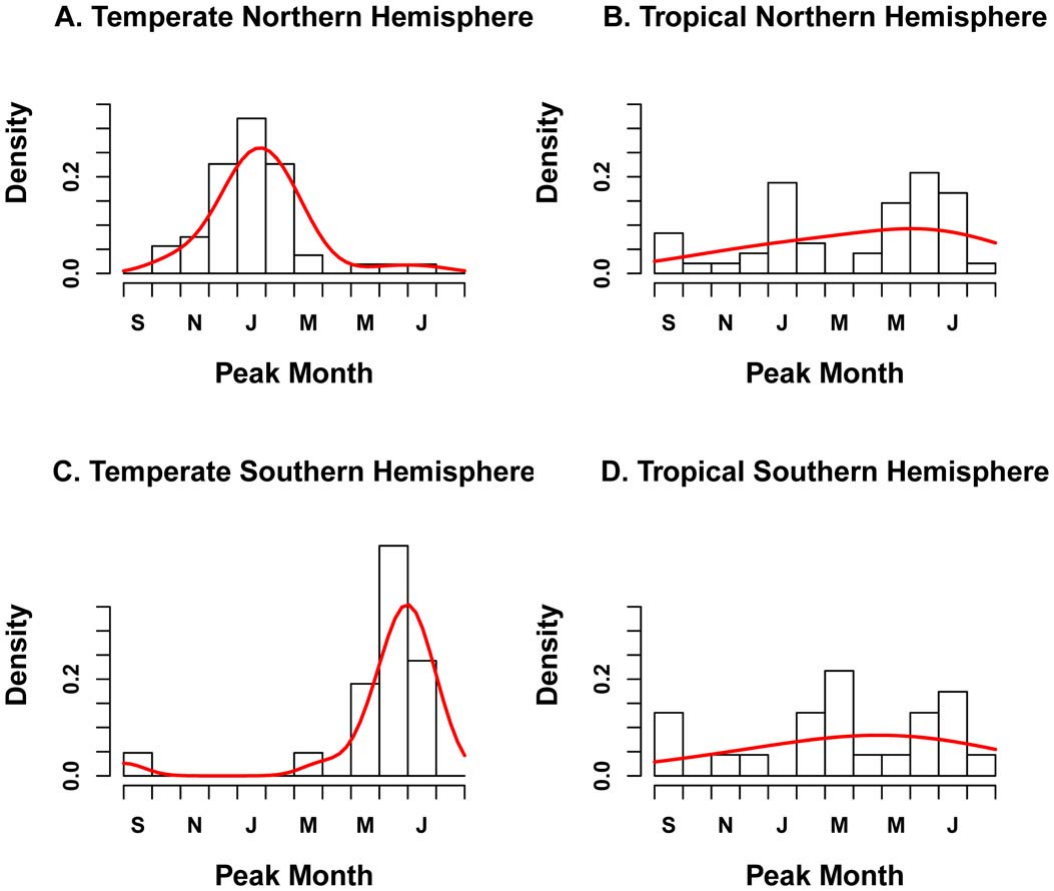
Distribution of RSV peak month by geographic zone (n = 96 locations). The black histogram represents observations while the red curve illustrates the fit of a Gaussian density kernel.

Peak RSV timing was highly focused on winter months in temperate locations of the Northern Hemisphere with a mode in February (n = 64 distinct locations, Table 2, Figure 4). The distribution of peak months was broader than that of influenza, with 14 locations (22%) reporting peaks outside the December-March winter period (Figure 5A). In particular, several multiyear studies reported biennial cycles of RSV activity, with peaks occurring every other year in the spring (April-May) in Stockholm, Sweden (59.3°N) and Turku, Finland (64.0°N) [42], [43]. In addition, two independent studies set in Taiwan (25.0°N) reported multiple RSV epidemics within the year, with peaks in April-March, July and October [33], [44].

In the tropical zone, RSV peak timing was more diverse than in temperate regions (Table 2, Figure 5B–D; n = 44). There was no difference in distribution of seasonal peaks between tropical regions of the Southern and Northern Hemispheres (Wilcoxon P = 0.26). Semi-annual peaks of RSV activity were identified in Hong Kong, China; Singapore; Kuala Lumpur, Malaysia; and Medellin, Colombia [45]–[49] (Figure 4).

Limited RSV information was available from temperate locations in the Southern Hemisphere (n = 20 locations). RSV epidemic peaks were clustered primarily in the winter months in this region, with a July mode (Table 2, Figure 5C). There were 4 outliers identified from relatively short prospective studies, reporting RSV peaks in October in Alice Springs, Australia (23.7°S) and in March-April in Cape Town, South Africa (33.9°S); Manhica, Mozambique (25.4°S); and Sao Paulo, Brazil (23.5°S) [50]–[53].

There was a weak association between RSV peak timing and latitude in both the Northern and Southern Hemispheres, with peak activity occurring gradually later in the year as absolute latitude increased (Table 3 and Figure S2; P ≤ 0.003). The relationship was robust to weighting observations by the no. of sample tested (Figure S2). There was no association between RSV peak timing, sample size, study settings or socio-economic factors.

#### 3.2. RSV Epidemic Duration

Median RSV epidemic duration was 5 months using a 5% epidemic threshold, with an interquartile range of 4–6 months, very similar to that of influenza (Wilcoxon P-value for difference between influenza and RSV = 0.67). In contrast to influenza, there was no difference in RSV epidemic duration between tropical and temperate locations (P = 0.66; Table 2). Additionally, there was no association between RSV epidemic duration and absolute latitude in either hemisphere or subregion (p>0.13; Table 3 and Figure S3). Weighting observations by the no. of specimen tested did not produce stronger patterns of association between RSV duration and latitude.

Study characteristics did not affect RSV epidemic duration (P≥0.12). As with influenza, a more conservative definition of epidemic threshold produced substantially lower duration estimates (median = 3 mo, IQR = 2–4 months), but confirmed the lack of association between RSV epidemic duration and geography or sampling intensity (Figure S3). Further sensitivity analyses limited to a subset of RSV studies conducted for over 2 years confirmed our main findings (Text S1 and Tables S2 and S3).

### 4. Comparison of Sub-national Studies Providing Information on Influenza and RSV

To explore potential differences in seasonal characteristics between influenza and RSV activity in the same location, controlling for study settings, we analyzed 28 studies providing information on both influenza and RSV. On average, the absolute difference between timing of peak influenza and RSV activity was 2.6 months, with influenza predating RSV circulation in 12 studies (43%). Peak timing differed by ≤ 1 month in 10 of these studies (35%). There was limited variation in duration of viral circulation by hemisphere or by virus (absolute variation less than 1.25 month on average, n = 16). Using the 10% epidemic threshold did not reveal further differences.

### 5. Time Series Models Fitted to Weekly National Surveillance Data from FluNet

The median length of the 85 country-specific time series meeting our minimum sample size criteria was 9 yrs (range 3–14), and the median number of positive specimens isolated per single respiratory season and country was 453 (range 53–25,050).

**Figure 6:**
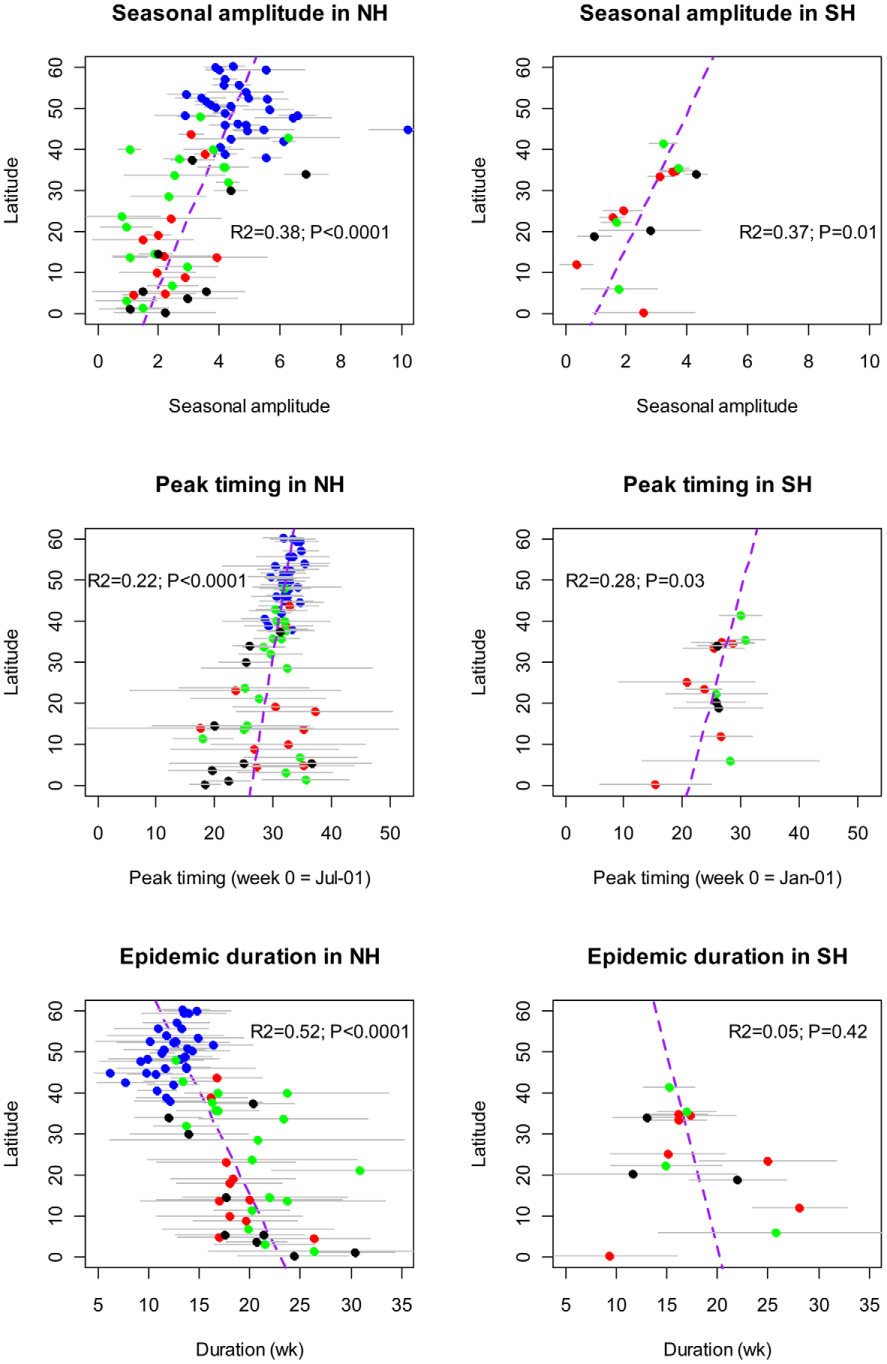
Latitudinal gradients in the seasonal amplitude, timing, and duration of influenza epidemics, based on time series modeling of weekly influenza virus surveillance data from 85 countries reporting to FluNet. Analysis was limited to countries providing ≥30 viruses for ≥3 yrs. Different colors represent different continents (blue = Europe; red = America; green = Asia-Pacific; black = Africa). Horizontal grey bars represent estimation error (top panels) or between-year fluctuation in seasonal characteristics (bottom panels, ±2 standard deviations).

Results of the time series analysis broadly confirmed the influenza patterns seen in the more highly resolved spatial data. In particular we found latitudinal gradients in influenza seasonal amplitude, peak timing, and epidemic duration in the Northern and Southern Hemispheres (Figure 6). Seasonal amplitude increased away from the Equator, and this was true in both Hemispheres (R^2^ = 0.37–0.38, P≤0.01, Fig. 6 top panels). Peak influenza timing occurred increasingly later in the season at higher latitudes (R^2^ = 0.22–0.28, P≤0.03, Fig. 6 middle panels), while low latitude countries experienced more between-year variability in timing (P<0.05).

Influenza epidemic duration increased at lower latitudes in the Northern Hemisphere (R^2^ = 0.52; P<0.0001, Fig. 6 bottom panels), although there was no association in the Southern Hemisphere. Overall, the median epidemic duration was 21 wks in tropical countries and 14 wks in temperate countries (Wilcoxon test, P<0.0001). Interestingly, between-year variability in epidemic duration was higher at lower latitudes (P<0.0001) and this association did not vary with the number of viruses isolated. Patterns were similar when using a higher threshold for epidemic activity. Further, geographical differences in timing, seasonal amplitude, and duration of epidemics were robust to the analysis of a subset of countries with denser sampling (78 countries reporting ≥100 positive specimens during ≥3 respiratory seasons).

## Discussion

To our knowledge, this is the first quantitative study to systematically review influenza and RSV seasonal patterns on a global scale. Due to strengthened respiratory virus surveillance in recent years, seasonality information was available for 137 distinct locations located on 5 continents, with one-third of our data representing the tropics. Further, systematic weekly influenza virus surveillance available from the WHO surveillance system FluNet allowed more refined time series modeling in a subset of 85 countries.

Our study suggests that the global seasonal patterns of influenza and RSV are broadly similar, with temperate locations of the Northern and Southern Hemisphere characterized by focused peaks of activity during their respective winters, and a wide range in the timing and duration of epidemics in the tropics. We found evidence challenging the common notion that these respiratory viruses are prevalent year-round in the tropics. In fact, 80% of tropical locations included our review experienced distinct RSV seasons lasting 6 months or less. Similarly, 50% of tropical locations in our sample experienced distinct influenza season. Both viruses exhibited weak latitudinal gradients in the timing of epidemics by Hemisphere, with peak timing occurring later in the respiratory season with increasing latitude. Time series models applied to FluNet data confirmed the presence of latitudinal gradients in influenza seasonal parameters, including peak timing, seasonal amplitude, epidemic duration, and between-year fluctuations in seasonal patterns.

Our joint analysis of influenza and RSV revealed notable differences between the two viruses. Specifically, RSV had broader distribution of peak timings, relative to that of influenza, even within the temperate zone, with more high latitude locations experiencing peak activity outside of typical winter months. Seasonal activity of the two viruses typically overlaps in the Western US [54] but not in the Netherlands or in the Southeastern US [10], [55]. In contrast to systematic regional variations in RSV timing in the US, influenza epidemics are well synchronized across this country [10], [56], suggesting differences in the spatial transmission of these viruses. It is generally thought that influenza does not persist over summer in temperate areas and that new virus variants emerge periodically from a source location presumably located in the tropics and spread globally [57]–[59]. These global viral migrations could explain the highly synchronized winter epidemics reported in the US, and between the US and Europe [56], [60]. In contrast, more localized or geographically diverse environmental factors could affect the dynamics of RSV epidemics [6], as suggested by seasonal differences in viral activity at small geographical scales [10], [61], [62].

Although the majority of tropical and temperate locations in our database experienced distinct respiratory virus seasons with epidemic duration lasting 6 months or less, it is worth commenting on the several outliers characterized by semi-annual viral peaks. Semi-annual influenza peaks were relatively common in South-East Asia, including in Bhaktapur, Nepal; Bangkok, Thailand; Manila, Philippines; Singapore; Hanoi, Vietnam; Hong Kong, China; Taiwan; and Okinawa, Japan [31], [35]–[41]. Predominance of semi-annual influenza activity, combined with particularly high population density and/or strong connectivity between subpopulations, could favor the emergence of new virus variants in this region [59]. We cannot rule out, however, the existence of additional locations experiencing semi-annual peaks of activity in undersampled regions, especially tropical areas of Africa, as only 6% of our study sites were located in this region. Thus, additional epidemiological and genetic data from understudied tropical regions is needed to fully elucidate the global circulation of influenza [63], [64].

While semi-annual seasonal patterns were less common for RSV than for influenza in our data, we identified 5 subtropical locations with semi-annual RSV peaks, including Taiwan, Hong Kong, Singapore, Malaysia, and Colombia [45]–[49]. We also noted multi-year periodicities of RSV epidemics in Northern Europe, where large winter epidemics are followed by milder outbreaks in the spring of the following year [42], [43], [65]. These multiyear cycles may be linked to shifts in the predominance of RSV A and B serotypes, although this hypothesis remains debated [66], [67]. Greater variability in the distribution of RSV timing in temperate areas, relative to that of influenza, could be attributable in part to these multiyear cycles.

The latitudinal gradients in influenza timing, duration, and seasonal amplitude evidenced in our study are reminiscent of previous reports from Brazil, Europe and Canada showing similar patterns at smaller geographic scales [68]–[72]. Our study also suggests that low-latitude settings experience more between-year variability in epidemic timing and duration than temperate locations. Whether these seasonal gradients are associated with differences in influenza annual attack rates remains an area of great interest for future research. Excess mortality studies have suggested that the 2009 pandemic impact was attenuated in Equatorial regions of Brazil as compared with the more temperate southern regions [73]; however, whether inter-pandemic influenza follows the same pattern remains unclear. Mortality studies only provide a partial answer as influenza-related mortality rates are affected by underlying health and socio-economic disparities, unrelated to influenza transmission [74]. Further, estimates of influenza effective reproduction number were slightly lower in tropical Brazil than in the US, hinting at geographical differences in influenza transmission dynamics [75]. Future modeling studies could address geographical differences in influenza reproduction number in a more systematic manner, although reliable morbidity data are lacking in many countries. Systematic studies conducted in parallel in distinct locations would be particularly useful to address differences in transmission dynamics, including serologic surveys and prospective follow-up of acute respiratory illnesses combined with laboratory testing.

In evaluating the accuracy of our results we note that this review is necessarily limited by the use of data from a variety of studies with different protocols. In order to provide a global picture of the virus patterns we needed to be fairly inclusive, and some of the studies included had small sample size and/or short duration, potentially biasing our analyses as timing may vary substantially between years. Fortunately, our sensitivity analyses suggest that sample size and study setting had little impact on the epidemic patterns and characteristics– peak time and duration–identified in this analysis. Further, we provided more refined time series analyses for a subset of countries providing systematic multi-year information to FluNet, solidifying our influenza results. Unfortunately, a similar global surveillance resource does not exist for RSV, hampering more refined seasonality analyses of this virus on a global scale. Overall, the lack of data from certain regions, such as Africa and the Southern Hemisphere, limited our ability to fully describe global patterns. Finally, we could not address potential differences in seasonality patterns between viral (sub)types of influenza and RSV. Building on previous work [68], further research making use of more precise time series data should focus on describing the effect of geography on the co-circulation of different subtypes, as well as the impact of antigenic change on viral seasonality patterns.

By providing a descriptive account of influenza and respiratory syncytial virus activity worldwide, this review is intended to support future research into the mechanisms underlying the patterns of transmission. In particular, a variety of climatic, population and geographic factors could be tested against the latitudinal gradients in seasonality of viral epidemics evidenced here. Our results for countries with published data go against the traditional concept that these viruses have year-round activity throughout the tropics. Such rationale likely stems from older studies that examined broad regions, and/or observed variable peaks throughout the year in smaller studies, without examining localized patterns in specific regions [2]. Given that most regions of the world experience influenza and RSV activity over a 3–5 months period–with the exception of parts of Asia and Central America –public health interventions need to be timed according to the local epidemic pattern of these viruses. Laboratory capacity in middle and low-income regions should be strengthened to optimize timing of influenza vaccine delivery and RSV prophylaxis.

## Supporting Information

Figure S1 **Flow Diagram of studies identified, scanned and included in the review of influenza and RSV seasonal activity.** Additional influenza information was retrieved from electronic surveillance websites (see main text).
(DOC)

Figure S2 **Regression of peak timing of activity against latitude, by virus and hemisphere.** Black and red dots represent all studies, with black lines representing ordinary least squares regression results (see Table 3 for coefficients and P-values). Red dots are for studies providing the no. of samples tested. No. samples tested was used in weighted least squares regression, indicated by red dashed lines (all p<0.03). These results indicate a weak latitudinal gradient in timing of peak virus activity, which is generally stronger in weighted regression.
(TIF)

Figure S3 **Regression of epidemic duration against latitude, by virus and hemisphere.** Red dots represent duration estimates based on the 10% threshold; red lines represent a least square regression linear model fit. Black dots represent duration estimates based on the 5% threshold; red lines represent a least square regression linear model fit (see Table 3 for estimates). Green lines represent the fit of a least square regression weighted by no. of samples tested.
(TIF)

Table S1 **List of studies and locations included in the review indicating the abbreviated reference, country and location, study duration and years, and type of information (influenza, RSV, both).** Table is sorted by descending latitude. A single study can appear several times if disaggregated information for multiple locations was reported. If a study covered a region rather than a city, or for multicenter studies that did not provide disaggregated data, we used the mean geographical location of study sites when available, or the country/regional capital, as indicated in parentheses in the Location(City, Region, State) column.
(XLS)

Table S2 **Sensitivity analysis: influenza and respiratory syncytial virus (RSV) peak timing and epidemic duration by geographic zone, limited to studies conducted for 2 years of more.**
(DOC)

Table S3 **Sensitivity analysis: association between absolute latitude, peak timing and duration of influenza and respiratory syncytial virus (RSV) epidemics, by geographic zone.** Analysis is limited to studies conducted for 2 years of more. Statistically significant P-values appear in bold.
(DOC)

Text S1 (DOC)
