# Supplementary material for: Latitudinal Variations in Seasonal Activity of Influenza and Respiratory Syncytial Virus (RSV): A Global Comparative Review
Source: PLoS One. 2013 Feb 14;8(2):e54445. doi: 10.1371/journal.pone.0054445 (PMC3573019; doi:10.1371/journal.pone.0054445)
Supplement: Figure S1 — Flow Diagram of studies identified, scanned and included in the review of influenza and RSV seasonal activity. Additional influenza information was retrieved from electronic surveillance websites (see main text). (DOC) [file pone.0054445.s001.doc]

**Supplementary Figure 1: Flow Diagram of studies identified, scanned and included in the review of influenza and RSV seasonal activity. Additional influenza information was retrieved from electronic surveillance websites (see main text).**

**Screening**

**Included**

**Eligibility**

**Identification**

Articles identified through PubMed search
(n = 1,560)

Additional articles identified through other sources (eg, reference lists of included papers) (n = 82 )

Titles and abstracts screened
(n = 1,642 )

Articles excluded (n = 931 )

- Article focused on animal or pandemic influenza

- No influenza or RSV laboratory-confirmed data presented

- Experimental study

- Surveillance covered less than 1 year

Full-text articles assessed for eligibility
(n = 711 )

Full-text articles excluded (n = 547)

- No laboratory-confirmed data

- Study focused on pandemic influenza

- Data duplicated information from (or was a subset of) another study

- Sample size criteria were not met (less than 24 flu or RSV specimens a year).

- No weekly or monthly viral activity data presented in a legible format

- Study was not conducted for ≥12 consecutive months.

Articles included in review
(n = 164 )
