# Supplementary figures and images for: Latitudinal Variations in Seasonal Activity of Influenza and Respiratory Syncytial Virus (RSV): A Global Comparative Review
Source: PLoS One. 2013 Feb 14;8(2):e54445. doi: 10.1371/journal.pone.0054445 (PMC3573019; doi:10.1371/journal.pone.0054445)

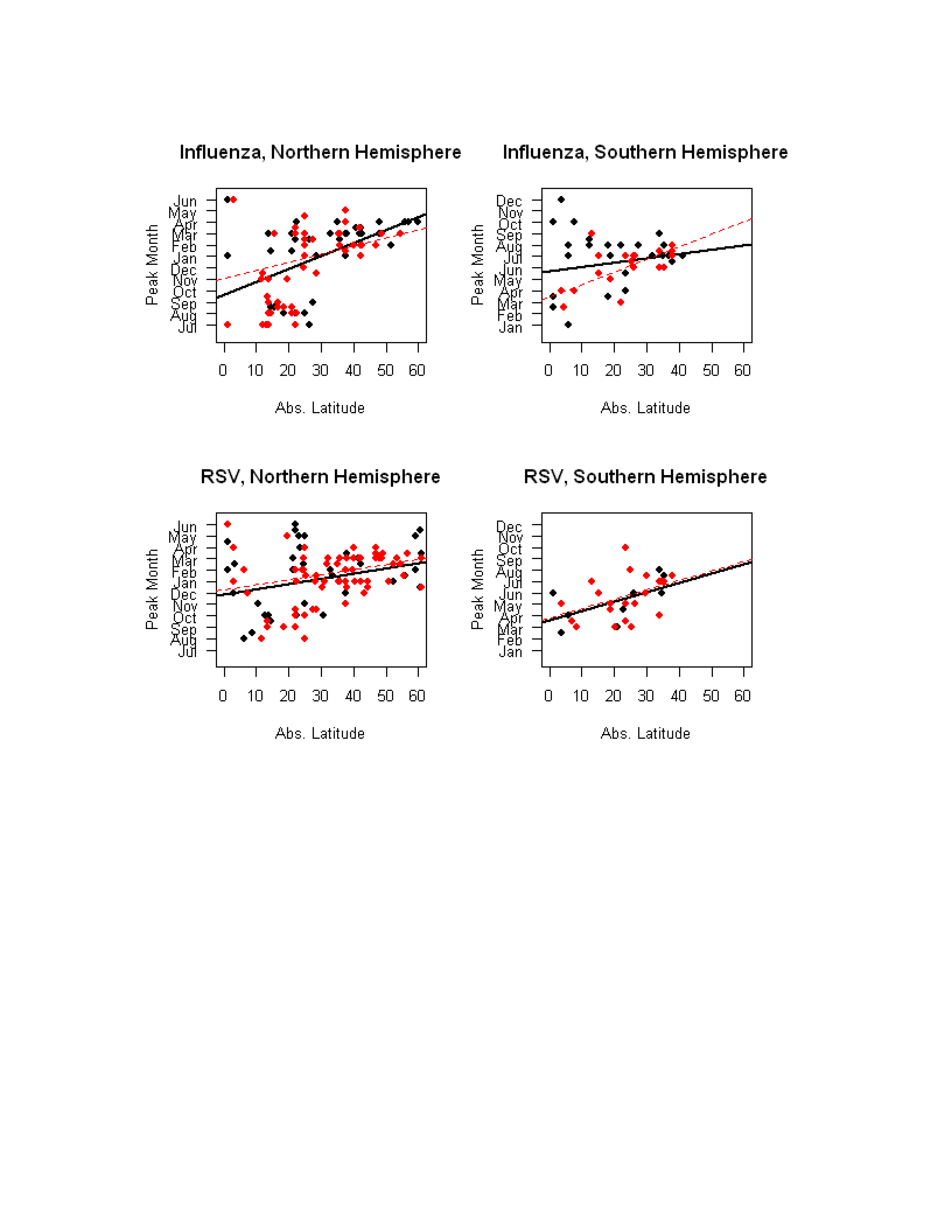

Supplement: Figure S2 — Regression of peak timing of activity against latitude, by virus and hemisphere. Black and red dots represent all studies, with black lines representing ordinary least squares regression results (see Table 3 for coefficients and P-values). Red dots are for studies providing the no. of samples tested. No. samples tested was used in weighted least squares regression, indicated by red dashed lines (all p<0.03). These results indicate a weak latitudinal gradient in timing of peak virus activity, which is generally stronger in weighted regression. (TIF) [file pone.0054445.s002.tif]

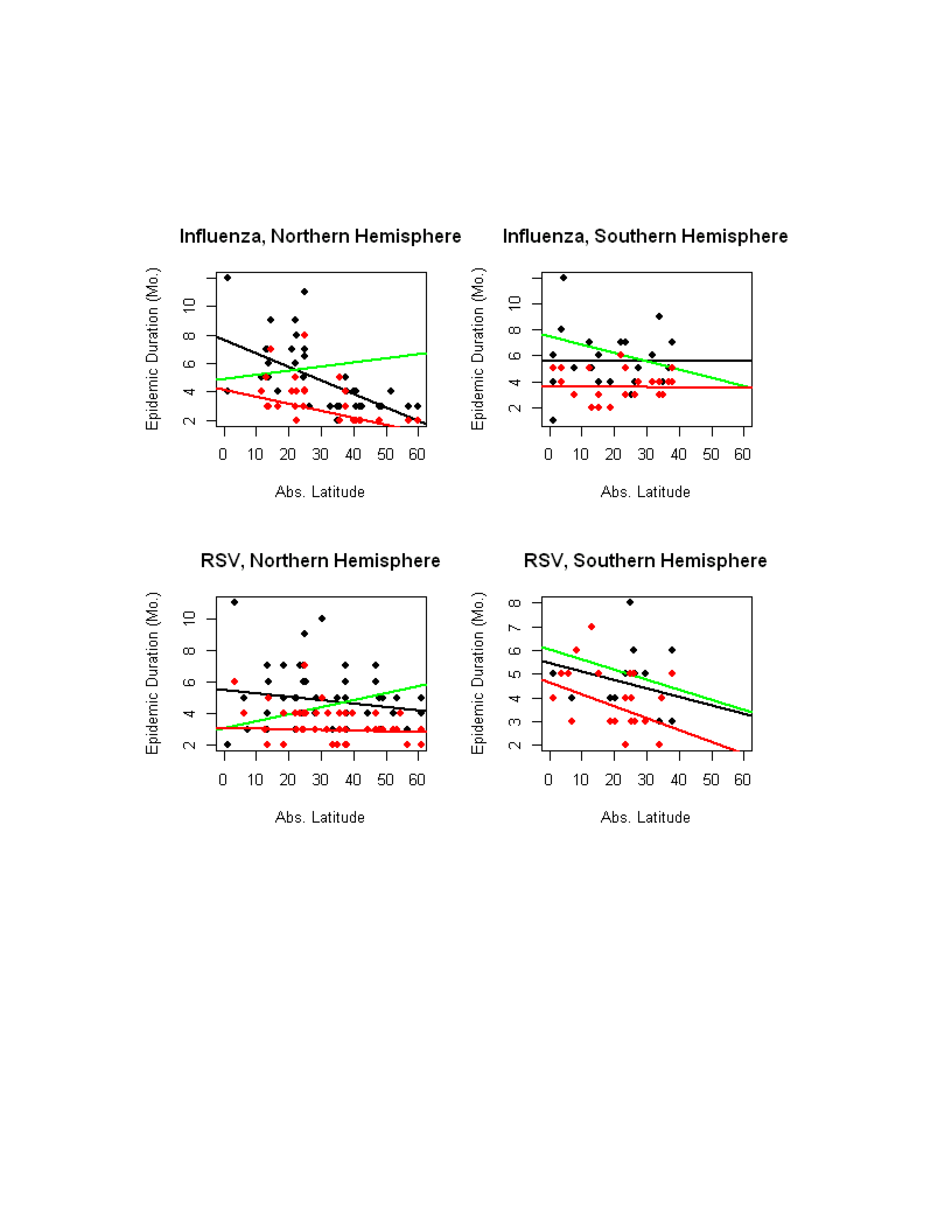

Supplement: Figure S3 — Regression of epidemic duration against latitude, by virus and hemisphere. Red dots represent duration estimates based on the 10% threshold; red lines represent a least square regression linear model fit. Black dots represent duration estimates based on the 5% threshold; red lines represent a least square regression linear model fit (see Table 3 for estimates). Green lines represent the fit of a least square regression weighted by no. of samples tested. (TIF) [file pone.0054445.s003.tif]
