# Supplementary material for: Latitudinal Variations in Seasonal Activity of Influenza and Respiratory Syncytial Virus (RSV): A Global Comparative Review
Source: PLoS One. 2013 Feb 14;8(2):e54445. doi: 10.1371/journal.pone.0054445 (PMC3573019; doi:10.1371/journal.pone.0054445)
Supplement: Table S2 — Sensitivity analysis: influenza and respiratory syncytial virus (RSV) peak timing and epidemic duration by geographic zone, limited to studies conducted for 2 years of more. (DOC) [file pone.0054445.s005.doc]

**Table S2:** Sensitivity analysis: influenza and respiratory syncytial virus (RSV) peak timing and epidemic duration by geographic zone, limited to studies conducted for 2 years of more.

| **Region** | **Influenza** | | | **RSV** | | |
| --- | --- | --- | --- | --- | --- | --- |
|  | **Peak timing** | | **Duration* (mo)** | **Peak timing** | | **Duration* (mo)** |
|  | Mode | Median (IQR) | Median (IQR); [mean] | Mode | Median (IQR) | Median (IQR); [mean] |
| NH temperate | Feb | Feb (mid-Jan – Mar) | 3.5 (3-5); [4.3] | Feb | Jan (Dec – Feb) | 4 (4-6); [4.9] |
| NH tropics | Jul | Jun (Feb – Jul) | 5 (4.5-7); [5.7] | Nov | Jan (Oct – May) | 5 (3-6); [4.9] |
| SH tropics | Apr | Apr (Mar – Jun) | 6 (5-7); [6.4] | May | Apr (Mar – May) | 5 (4-5); [4.8] |
| SH temperate | Jul | Jul (Jun - Aug) | 5 (4.5-7); [5.6] | Jul | Jul (Jun – mid-Jul) | 3.5 (36); [4.0] |

IQR: Interquartile range; SH: Southern Hemisphere; NH: Northern Hemisphere

This table provides information on the number of distinct studies included in the review, while information on the number of distinct locations is provided in the text.

* Based on a 5% threshold; see methods for details
