# Supplementary material for: Latitudinal Variations in Seasonal Activity of Influenza and Respiratory Syncytial Virus (RSV): A Global Comparative Review
Source: PLoS One. 2013 Feb 14;8(2):e54445. doi: 10.1371/journal.pone.0054445 (PMC3573019; doi:10.1371/journal.pone.0054445)
Supplement: Table S3 — Sensitivity analysis: association between absolute latitude, peak timing and duration of influenza and respiratory syncytial virus (RSV) epidemics, by geographic zone. Analysis is limited to studies conducted for 2 years of more. Statistically significant P-values appear in bold. (DOC) [file pone.0054445.s006.doc]

**Table S3:** Sensitivity analysis: association between absolute latitude, peak timing and duration of influenza and respiratory syncytial virus (RSV) epidemics, by geographic zone. Analysis is limited to studies conducted for 2 years of more. Statistically significant P-values appear in bold.

| Epidemic duration ~ Latitude | | | | | | |
| --- | --- | --- | --- | --- | --- | --- |
|  | Influenza | | | RSV | | |
| Region | No. studies | Coefficient (R^2^) | P-value | No. studies | Coefficient (R^2^) | P-value |
| All regions | 54 | β = -0.07 (R^2^ = 0.16) | **P = 0.003** | 55 | β = -0.02 (R^2^ = 0.03) | P = 0.21 |
| NH | 32 | β = -0.08 (R^2^ = 0.19) | **P = 0.008** | 43 | β = -0.02 (R^2^ = 0.03) | P = 0.25 |
| SH | 22 | β = -0.04 (R^2^ = 0.06) | P = 0.28 | 12 | β = -0.04 (R^2^ =0.17) | P = 0.18 |
| Peak month ~ Latitude | | | | | | |
|  | Influenza | | | RSV | | |
| Region | No. studies | Coefficient (R^2^) | P-value | No. studies | Coefficient (R^2^) | P-value |
| NH | 68 | β = -0.007 (R^2^ = 0.001) | P = 0.81 | 75 | β = -0.02 (R^2^ = 0.01) | P = 0.39 |
| SH | 19 | β = -0.04 (R^2^ = 0.02) | P = 0.57 | 20 | β = 0.11 (R^2^ = 0.33) | **P = 0.008** |

SH: Southern Hemisphere; NH: Northern Hemisphere

This table provides information on the number of distinct studies included in the review, while information on the number of distinct locations is provided in the text.

Duration estimate based on a 5% threshold; see methods for details
