# Supplementary material for: Latitudinal Variations in Seasonal Activity of Influenza and Respiratory Syncytial Virus (RSV): A Global Comparative Review
Source: PLoS One. 2013 Feb 14;8(2):e54445. doi: 10.1371/journal.pone.0054445 (PMC3573019; doi:10.1371/journal.pone.0054445)
Supplement: Text S1 — (DOC) [file pone.0054445.s007.doc]

**Latitudinal variations in seasonal activity of influenza and respiratory syncytial virus (RSV): a global comparative review**

**Supplementary Information**

**Authors** Kimberly Bloom-Feshbach^1,2^, Wladimir J. Alonso^1^, Vivek Charu^1,,3^, James Tamerius^4^, Lone Simonsen^1,5^, Mark A. Miller^1^, Cécile Viboud^1^

**Sensitivity analyses of highly resolved spatial data restricted to longer studies**

To further test the robustness of our results to study duration, we conducted a sensitivity analysis restricted to studies lasting for 2 years of more, which consisted in 85 studies for influenza and 83 for RSV. Descriptive statistics of peak timing for influenza and RSV were broadly consistent with our analysis of the full data set, with peaks concentrated in winter months in temperate regions and greater diversity in the tropics (Table S2). The association between epidemic duration and latitude was also consistent with our main analysis, with a significant relationship for influenza, driven by the larger dataset from the Northern Hemisphere, whereby longer epidemics were preferentially found at lower latitudes (Table S3). In contrast, there was no association between duration and latitude for RSV, also in line with the main analysis. The association between peak timing and latitude was less pronounced in the more stringent dataset, compared to the main analysis (Table S3).

**Complete list of references of studies presented in Supplementary Table 1 (sorted alphabetically)**

1. Agrawal, A.S., et al., *Comparative evaluation of real-time PCR and conventional RT-PCR during a 2 year surveillance for influenza and respiratory syncytial virus among children with acute respiratory infections in Kolkata, India, reveals a distinct seasonality of infection.* J Med Microbiol, 2009. **58**(Pt 12): p. 1616-22.

2. Ahn, K.M., et al., *Clinical characteristics of acute viral lower respiratory tract infections in hospitalized children in Seoul, 1996-1998.* J Korean Med Sci, 1999. **14**(4): p. 405-11.

3. al Rashed, A., *Role of Mycoplasma pneumoniae in acute respiratory-tract infections in Saudi paediatric patients.* Ann Trop Med Parasitol, 1998. **92**(5): p. 595-601.

4. al-Hajjar, S., et al., *Respiratory viruses in children attending a major referral centre in Saudi Arabia.* Ann Trop Paediatr, 1998. **18**(2): p. 87-92.

5. Al-Toum, R., S. Bdour, and H. Ayyash, *Epidemiology and Clinical Characteristics of Respiratory Syncytial Virus Infections in Jordan.* J Trop Pediatr, 2006.

6. de Arruda, E., et al., *Acute respiratory viral infections in ambulatory children of urban northeast Brazil.* J Infect Dis, 1991. **164**(2): p. 252-8.

7. Artiles-Campelo, F., et al., *[Etiology of acute viral respiratory tract infections in children from Gran Canaria, the Canary Islands (Spain)].* Enferm Infecc Microbiol Clin, 2006. **24**(9): p. 556-61.

8. Bakir, T.M., M. Halawani, and S. Ramia, *Viral aetiology and epidemiology of acute respiratory infections in hospitalized Saudi children.* J Trop Pediatr, 1998. **44**(2): p. 100-3.

9. Bdour, S., *Respiratory syncytial virus subgroup A in hospitalized children in Zarqa, Jordan.* Ann Trop Paediatr, 2001. **21**(3): p. 253-61.

10. Bedoya, V.I., V. Abad, and H. Trujillo, *Frequency of respiratory syncytial virus in hospitalized infants with lower acute respiratory tract infection in Colombia.* Pediatr Infect Dis J, 1996. **15**(12): p. 1123-4.

11. Bellei, N., et al., *Acute respiratory infection and influenza-like illness viral etiologies in Brazilian adults.* J Med Virol, 2008. **80**(10): p. 1824-7.

12. Berlioz-Arthaud, A. and I.G. Barr, *Laboratory-based influenza surveillance in New Caledonia, 1999-2003.* Trans R Soc Trop Med Hyg, 2005. **99**(4): p. 290-300.

13. Berner, R., et al., *Community and nosocomially acquired respiratory syncytial virus infection in a German paediatric hospital from 1988 to 1999.* Eur J Pediatr, 2001. **160**(9): p. 541-7.

14. Bharaj, P., et al., *Respiratory viral infections detected by multiplex PCR among pediatric patients with lower respiratory tract infections seen at an urban hospital in Delhi from 2005 to 2007.* Virol J, 2009. **6**: p. 89.

15. Blair, P.J., et al., *Influenza epidemiology and characterization of influenza viruses in patients seeking treatment for acute fever in Cambodia.* Epidemiol Infect, 2010. **138**(2): p. 199-209.

16. Bonroy, C., et al., *Use of a multiplex real-time PCR to study the incidence of human metapneumovirus and human respiratory syncytial virus infections during two winter seasons in a Belgian paediatric hospital.* Clin Microbiol Infect, 2007. **13**(5): p. 504-9.

17. Bosch Ferrer, A., et al., *[Epidemiology of respiratory syncytial virus infections in the Canary Islands].* An Esp Pediatr, 1992. **36**(4): p. 298-300.

18. Bosso, P.A.R., et al., *Human respiratory syncytial virus detection in children admitted at a community hospital in Botucatu, SP, Brazil.* Brazilian Journal of Microbiology, 2004. **35**: p. 348-351.

19. Bourgeois, F.T., et al., *Validation of syndromic surveillance for respiratory infections.* Ann Emerg Med, 2006. **47**(3): p. 265 e1.

20. Cabello, C., et al., *Frequency of viruses associated with acute respiratory infections in children younger than five years of age at a locality of Mexico City.* Mem Inst Oswaldo Cruz, 2006. **101**(1): p. 21-4.

21. Calegari, T., et al., *Clinical-epidemiological evaluation of respiratory syncytial virus infection in children attended in a public hospital in midwestern Brazil.* Braz J Infect Dis, 2005. **9**(2): p. 156-61.

22. Carballal, G., et al., *Multicentered study of viral acute lower respiratory infections in children from four cities of Argentina, 1993-1994.* J Med Virol, 2001. **64**(2): p. 167-74.

23. http://www.cdc.gov/flu/weekly/fluactivity.htm, *CDC / World Health Organization and National Respiratory and Enteric Virus Surveillance System.* Accessed July 1, 2012.

24. Chan, P.K., et al., *Epidemiology of respiratory syncytial virus infection among paediatric patients in Hong Kong: seasonality and disease impact.* Epidemiol Infect, 1999. **123**(2): p. 257-62.

25. Chan, P.K., et al., *Seasonal influenza activity in Hong Kong and its association with meteorological variations.* J Med Virol, 2009. **81**(10): p. 1797-806.

26. Chan, P.W., et al., *Seasonal variation in respiratory syncytial virus chest infection in the tropics.* Pediatr Pulmonol, 2002. **34**(1): p. 47-51.

27. Chatterjee, S., et al., *A study of influenza A virus in the city of Calcutta, India, high lighting the strain prevalence.* Acta Microbiol Pol, 1996. **45**(3-4): p. 279-83.

28. Checon, R.E., et al., *Short report: seasonal pattern of respiratory syncytial virus in a region with a tropical climate in southeastern Brazil.* Am J Trop Med Hyg, 2002. **67**(5): p. 490-1.

29. Chew, F.T., et al., *Seasonal trends of viral respiratory tract infections in the tropics.* Epidemiol Infect, 1998. **121**(1): p. 121-8.

30. Chiu, S.S., et al., *Influenza-related hospitalizations among children in Hong Kong.* N Engl J Med, 2002. **347**(26): p. 2097-103.

31. Chiu, S.S., et al., *Virologically confirmed population-based burden of hospitalization caused by influenza A and B among children in Hong Kong.* Clin Infect Dis, 2009. **49**(7): p. 1016-21.

32. Choi, Eun H., et al., *The Association of Newly Identified Respiratory Viruses with Lower Respiratory Tract Infections in Korean Children, 2000–2005.* Clinical Infectious Diseases, 2006. **43**(5): p. 585-592.

33. Chutinimitkul, S., et al., *Molecular characterization and phylogenetic analysis of H1N1 and H3N2 human influenza A viruses among infants and children in Thailand.* Virus Res, 2008. **132**(1-2): p. 122-31.

34. Costa, L.F., et al., *Respiratory viruses in children younger than five years old with acute respiratory disease from 2001 to 2004 in Uberlandia, MG, Brazil.* Mem Inst Oswaldo Cruz, 2006. **101**(3): p. 301-6.

35. Dapat, C., et al., *Epidemiology of human influenza A and B viruses in Myanmar from 2005 to 2007.* Intervirology, 2009. **52**(6): p. 310-20.

36. de Mello, W.A., et al., *The dilemma of influenza vaccine recommendations when applied to the tropics: the Brazilian case examined under alternative scenarios.* PLoS One, 2009. **4**(4): p. e5095.

37. Djelantik, I.G., et al., *Incidence and clinical features of hospitalization because of respiratory syncytial virus lower respiratory illness among children less than two years of age in a rural Asian setting.* Pediatr Infect Dis J, 2003. **22**(2): p. 150-7.

38. Dosseh, A., et al., *Epidemiological and virological influenza survey in Dakar, Senegal: 1996-1998.* Am J Trop Med Hyg, 2000. **62**(5): p. 639-43.

39. Dowell, S.F., et al., *Respiratory syncytial virus is an important cause of community-acquired lower respiratory infection among hospitalized adults.* J Infect Dis, 1996. **174**(3): p. 456-62.

40. Druce, J., et al., *Laboratory diagnosis and surveillance of human respiratory viruses by PCR in Victoria, Australia, 2002-2003.* J Med Virol, 2005. **75**(1): p. 122-9.

41. Eriksson, M., et al., *Population-based rates of severe respiratory syncytial virus infection in children with and without risk factors, and outcome in a tertiary care setting.* Acta Paediatr, 2002. **91**(5): p. 593-8.

42. http://www.eiss.org/index.cgi, *European Influenza Surveillance Scheme (EISS).* Accessed July 1, 2012.

43. Fe, M.M., A.J. Monteiro, and F.E. Moura, *Parainfluenza virus infections in a tropical city: clinical and epidemiological aspects.* Braz J Infect Dis, 2008. **12**(3): p. 192-7.

44. Firestone, S.M., et al., *Annual report of the National Influenza Surveillance Scheme, 2005.* Commun Dis Intell, 2006. **30**(2): p. 189-200.

45. FluNet. 2000-2006.

46. Fodha, I., et al., *Epidemiological and antigenic analysis of respiratory syncytial virus in hospitalised Tunisian children, from 2000 to 2002.* J Med Virol, 2004. **72**(4): p. 683-7.

47. Fuller, D.G., et al., *Analysis of respiratory viral coinfection and cytomegalovirus coisolation in pediatric inpatients.* Pediatr Infect Dis J, 2005. **24**(3): p. 195-200.

48. Gaglani, M.J., et al., *Direct and total effectiveness of the intranasal, live-attenuated, trivalent cold-adapted influenza virus vaccine against the 2000-2001 influenza A(H1N1) and B epidemic in healthy children.* Arch Pediatr Adolesc Med, 2004. **158**(1): p. 65-73.

49. Garcia-Garcia, M.L., et al., *Human metapneumovirus infections in hospitalised infants in Spain.* Arch Dis Child, 2006. **91**(4): p. 290-5.

50. Gordon, A., et al., *Prevalence and seasonality of influenza-like illness in children, Nicaragua, 2005-2007.* Emerg Infect Dis, 2009. **15**(3): p. 408-14.

51. Halstead, D.C. and S.G. Jenkins, *Continuous non-seasonal epidemic of respiratory syncytial virus infection in the southeast United States.* South Med J, 1998. **91**(5): p. 433-6.

52. Hampson, A.W., *Epidemiological data on influenza in Asian countries.* Vaccine, 1999. **17 Suppl 1**: p. S19-23.

53. Hasegawa, G., et al., *Influenza virus infections in Yangon, Myanmar.* J Clin Virol, 2006. **37**(3): p. 233-4.

54. Huang, Y.C., et al., *Epidemiology of respiratory syncytial virus infection among paediatric inpatients in northern Taiwan.* Eur J Pediatr, 2001. **160**(9): p. 581-2.

55. Hussey, G.D., et al., *Respiratory syncytial virus infection in children hospitalised with acute lower respiratory tract infection.* S Afr Med J, 2000. **90**(5): p. 509-12.

56. http://idsc.nih.go.jp/index.html, *Japan’s Infectious Disease Surveillance Center.* Accessed July 1, 2012.

57. Irmen, K.E. and J.J. Kelleher, *Use of monoclonal antibodies for rapid diagnosis of respiratory viruses in a community hospital.* Clin Diagn Lab Immunol, 2000. **7**(3): p. 396-403.

58. Iwane, M.K., et al., *Population-based surveillance for hospitalizations associated with respiratory syncytial virus, influenza virus, and parainfluenza viruses among young children.* Pediatrics, 2004. **113**(6): p. 1758-64.

59. Jennings, L.C., *Influenza surveillance in New Zealand.* Vaccine, 1999. **17 Suppl 1**: p. S115-7.

60. Jian, J.W., et al., *Genetic and epidemiological analysis of influenza virus epidemics in Taiwan during 2003 to 2006.* J Clin Microbiol, 2008. **46**(4): p. 1426-34.

61. Johnson, A.W., et al., *Etiologic agents and outcome determinants of community-acquired pneumonia in urban children: a hospital-based study.* J Natl Med Assoc, 2008. **100**(4): p. 370-85.

62. Kaneko, M., et al., *Impact of respiratory syncytial virus infection as a cause of lower respiratory tract infection in children younger than 3 years of age in Japan.* J Infect, 2002. **44**(4): p. 240-3.

63. Kanra, G., S. Tezcan, and G. Yilmaz, *Respiratory syncytial virus epidemiology in Turkey.* Turk J Pediatr, 2005. **47**(4): p. 303-8.

64. Karron, R.A., et al., *Severe respiratory syncytial virus disease in Alaska native children. RSV Alaska Study Group.* J Infect Dis, 1999. **180**(1): p. 41-9.

65. Kelly, H., et al., *Laboratory-supported influenza surveillance in Victorian sentinel general practices.* Commun Dis Intell, 2000. **24**(12): p. 379-83.

66. Kim, M.R., H.R. Lee, and G.M. Lee, *Epidemiology of acute viral respiratory tract infections in Korean children.* J Infect, 2000. **41**(2): p. 152-8.

67. Koetz, A., et al., *Detection of human coronavirus NL63, human metapneumovirus and respiratory syncytial virus in children with respiratory tract infections in south-west Sweden.* Clin Microbiol Infect, 2006. **12**(11): p. 1089-96.

68. Lagos, R., L.F. Avendano, and M.M. Levine, *[Systematic surveillance of influenza, syncytial respiratory, parainfluenza and adenovirus in children with acute respiratory infections].* Rev Med Chil, 1999. **127**(9): p. 1063-72.

69. Laguna-Torres, V.A., et al., *Influenza-Like Illness Sentinel Surveillance in Peru.* PLoS One, 2009. **4**(7): p. e6118.

70. Law, B.J., X. Carbonell-Estrany, and E.A. Simoes, *An update on respiratory syncytial virus epidemiology: a developed country perspective.* Respir Med, 2002. **96 Suppl B**: p. S1-7.

71. Lee, W.K. and B.W. Young, *Infectious diseases in children admitted from a residential child care centre.* Hong Kong Med J, 2006. **12**(2): p. 119-24.

72. Lee, J.T., et al., *Epidemiology of respiratory syncytial virus infection in northern Taiwan, 2001-2005 -- seasonality, clinical characteristics, and disease burden.* J Microbiol Immunol Infect, 2007. **40**(4): p. 293-301.

73. Lee, V.J., et al., *Influenza excess mortality from 1950-2000 in tropical Singapore.* PLoS One, 2009. **4**(12): p. e8096.

74. Li, C.K., B.C. Choi, and T.W. Wong, *Influenza-related deaths and hospitalizations in Hong Kong: a subtropical area.* Public Health, 2006. **120**(6): p. 517-24.

75. Light, M., *Respiratory syncytial virus seasonality in southeast Florida: results from three area hospitals caring for children.* Pediatr Infect Dis J, 2007. **26**(11 Suppl): p. S55-9.

76. Loscertales, M.P., et al., *Epidemiology and clinical presentation of respiratory syncytial virus infection in a rural area of southern Mozambique.* Pediatr Infect Dis J, 2002. **21**(2): p. 148-55.

77. Madhi, S.A., et al., *Increased burden of respiratory viral associated severe lower respiratory tract infections in children infected with human immunodeficiency virus type-1.* J Pediatr, 2000. **137**(1): p. 78-84.

78. Madhi, S.A., et al., *Respiratory syncytial virus associated illness in high-risk children and national characterisation of the circulating virus genotype in South Africa.* J Clin Virol, 2003. **27**(2): p. 180-9.

79. Maitreyi, R.S., et al., *Rapid detection of respiratory viruses by centrifugation enhanced cultures from children with acute lower respiratory tract infections.* J Clin Virol, 2000. **16**(1): p. 41-7.

80. Mardy, S., et al., *Influenza activity in Cambodia during 2006-2008.* BMC Infect Dis, 2009. **9**: p. 168.

81. Mathisen, M., et al., *RNA viruses in community-acquired childhood pneumonia in semi-urban Nepal; a cross-sectional study.* BMC Med, 2009. **7**: p. 35.

82. McAdam, A.J., et al., *Human metapneumovirus in children tested at a tertiary-care hospital.* J Infect Dis, 2004. **190**(1): p. 20-6.

83. McAnerney, J.M., S. Johnson, and B.D. Schoub, *Surveillance of respiratory viruses. A 10-year laboratory-based study.* S Afr Med J, 1994. **84**(8 Pt 1): p. 473-7.

84. Medici, M.C., et al., *Four year incidence of respiratory syncytial virus infection in infants and young children referred to emergency departments for lower respiratory tract diseases in Italy: the "Osservatorio VRS" Study (2000-2004).* New Microbiol, 2006. **29**(1): p. 35-43.

85. Mizuta, K., et al., *Epidemiology of influenza virus infections in children with acute respiratory infections in Zambia.* Ann Trop Paediatr, 1997. **17**(2): p. 115-9.

86. Mokhtari-Azad, T., et al., *Influenza surveillance in the Islamic Republic of Iran from 1991 to 2001.* East Mediterr Health J, 2004. **10**(3): p. 315-21.

87. Monto, A.S., et al., *Medical practice-based influenza surveillance: viral prevalence and assessment of morbidity.* Am J Epidemiol, 1995. **141**(6): p. 502-6.

88. Moura, F.E., et al., *Respiratory syncytial virus infections during an epidemic period in Salvador, Brazil. Viral antigenic group analysis and description of clinical and epidemiological aspects.* Mem Inst Oswaldo Cruz, 2003. **98**(6): p. 739-43.

89. Moura, F.E., et al., *Respiratory syncytial virus infections in northeastern Brazil: seasonal trends and general aspects.* Am J Trop Med Hyg, 2006. **74**(1): p. 165-7.

90. Moura, F.E., A.C. Perdigao, and M.M. Siqueira, *Seasonality of influenza in the tropics: a distinct pattern in northeastern Brazil.* Am J Trop Med Hyg, 2009. **81**(1): p. 180-3.

91. Mulholland, E.K., et al., *Etiology of serious infections in young Gambian infants.* Pediatr Infect Dis J, 1999. **18**(10 Suppl): p. S35-41.

92. Mullins, J.A., et al., *Substantial variability in community respiratory syncytial virus season timing.* Pediatr Infect Dis J, 2003. **22**(10): p. 857-62.

93. Nascimento, J.P., et al., *Longitudinal study of acute respiratory diseases in Rio de Janeiro: occurrence of respiratory viruses during four consecutive years.* Rev Inst Med Trop Sao Paulo, 1991. **33**(4): p. 287-96.

94. http://www.health.gov.au/internet/wcms/publishing.nsf/Content/cda-pubs-annlrpt-fluannrep.htm, *Australian Government Department of Health and Ageing, Influenza - National Influenza Surveillance Scheme Annual reports.* Accessed July 1, 2012.

95. Nguyen, H.T., et al., *National influenza surveillance in Vietnam, 2006-2007.* Vaccine, 2009. **28**(2): p. 398-402.

96. Nguyen, H.L., et al., *Epidemiology of influenza in Hanoi, Vietnam, from 2001 to 2003.* J Infect, 2007. **55**(1): p. 58-63.

97. Nokes, D.J., et al., *Respiratory syncytial virus infection and disease in infants and young children observed from birth in Kilifi District, Kenya.* Clin Infect Dis, 2008. **46**(1): p. 50-7.

98. Noyola, D.E. and P.B. Mandeville, *Effect of climatological factors on respiratory syncytial virus epidemics.* Epidemiol Infect, 2008. **136**(10): p. 1328-32.

99. Noyola, D.E., et al., *Impact of respiratory syncytial virus on hospital admissions in children younger than 3 years of age.* J Infect, 2007. **54**(2): p. 180-4.

100. O'Kelly, E.A. and I.B. Hillary, *Epidemiology of respiratory syncytial virus infection among infants over three winter seasons.* Ir J Med Sci, 1991. **160**(1): p. 12-6.

101. Park, A.W. and K. Glass, *Dynamic patterns of avian and human influenza in east and southeast Asia.* Lancet Infect Dis, 2007. **7**(8): p. 543-8.

102. Pecchini, R., et al., *Incidence and clinical characteristics of the infection by the respiratory syncytial virus in children admitted in Santa Casa de Sao Paulo Hospital.* Braz J Infect Dis, 2008. **12**(6): p. 476-9.

103. Phillips, P.A., et al., *Viruses associated with acute lower respiratory tract infections in children from the eastern highlands of Papua New Guinea (1983-1985).* Southeast Asian J Trop Med Public Health, 1990. **21**(3): p. 373-82.

104. Rakoto-Andrianarivelo, M., et al., *[Influenza surveillance in Tananarive during the year 1992].* Arch Inst Pasteur Madagascar, 1993. **60**(1-2): p. 9-13.

105. Ramamurty, N., et al., *Influenza activity among the paediatric age group in Chennai.* Indian J Med Res, 2005. **121**(6): p. 776-9.

106. Rao, B.L. and K. Banerjee, *Influenza surveillance in Pune, India, 1978-90.* Bull World Health Organ, 1993. **71**(2): p. 177-81.

107. Rao, B.L., et al., *Influenza surveillance in Pune, India, 2003.* Southeast Asian J Trop Med Public Health, 2005. **36**(4): p. 906-9.

108. Reese, P.E. and N.J. Marchette, *Respiratory syncytial virus infection and prevalence of subgroups A and B in Hawaii.* J Clin Microbiol, 1991. **29**(11): p. 2614-5.

109. Resch, B., et al., *Epidemiology of respiratory syncytial virus infection in Southern Austria.* Pediatr Infect Dis J, 2000. **19**(6): p. 587-8.

110. Resch, B., W. Gusenleitner, and W. Muller, *The impact of respiratory syncytial virus infection: a prospective study in hospitalized infants younger than 2 years.* Infection, 2002. **30**(4): p. 193-7.

111. Robertson, S.E., et al., *Respiratory syncytial virus infection: denominator-based studies in Indonesia, Mozambique, Nigeria and South Africa.* Bull World Health Organ, 2004. **82**(12): p. 914-22.

112. Robinson, J.L., et al., *Seasonality and clinical features of human metapneumovirus infection in children in Northern Alberta.* Journal of Medical Virology, 2005. **76**(1): p. 98-105.

113. Rossi, G.A., M.C. Medici, and R. Merolla, *Incidence of respiratory syncytial virus positivity in young Italian children referred to the emergency departments for lower respiratory tract infection over two consecutive epidemic seasons.* Infection, 2005. **33**(1): p. 18-24.

114. Saijo, M., et al., *The role of respiratory syncytial virus in acute bronchiolitis in small children in northern Japan.* Acta Paediatr Jpn, 1994. **36**(4): p. 371-4.

115. Saijo, M., et al., *Respiratory syncytial virus infection in children with acute respiratory infections in Zambia.* Epidemiol Infect, 1998. **121**(2): p. 397-400.

116. Saijo, M., et al., *Respiratory syncytial virus infection in lower respiratory tract and asthma attack in hospitalized children in North Hokkaido, Japan.* Acta Paediatr Jpn, 1993. **35**(3): p. 233-7.

117. Saikusa, M., et al., *[An epidemiological study of respiratory syncytial virus infection in Yokohama, Japan for five years].* Kansenshogaku Zasshi, 2005. **79**(6): p. 381-7.

118. Sapin, G., A. Michault, and C. Simac, *[Seasonal trends of respiratory syncytial virus infections on Reunion Island gathering data among hospitalized children].* Bull Soc Pathol Exot, 2001. **94**(1): p. 3-4.

119. Sato, M., et al., *Molecular epidemiology of respiratory syncytial virus infections among children with acute respiratory symptoms in a community over three seasons.* J Clin Microbiol, 2005. **43**(1): p. 36-40.

120. Schoub, B.D., et al., *Benefits and limitations of the Witwatersrand influenza and acute respiratory infections surveillance programme.* S Afr Med J, 1994. **84**(10): p. 674-8.

121. Shih, S.R., et al., *Laboratory-based surveillance and molecular epidemiology of influenza virus in Taiwan.* J Clin Microbiol, 2005. **43**(4): p. 1651-61.

122. Simmerman, J.M., et al., *Incidence, seasonality and mortality associated with influenza pneumonia in Thailand: 2005-2008.* PLoS One, 2009. **4**(11): p. e7776.

123. Simmerman, J.M., et al., *The cost of influenza in Thailand.* Vaccine, 2006. **24**(20): p. 4417-26.

124. Simmerman, J.M., et al., *Influenza in Thailand: a case study for middle income countries.* Vaccine, 2004. **23**(2): p. 182-7.

125. Singleton, R.J., et al., *Hospitalizations for respiratory syncytial virus infection in Alaska Native children.* Pediatr Infect Dis J, 1995. **14**(1): p. 26-30.

126. Singleton, R.J., et al., *Decline in respiratory syncytial virus hospitalizations in a region with high hospitalization rates and prolonged season.* Pediatr Infect Dis J, 2006. **25**(12): p. 1116-22.

127. Singleton, R., et al., *RSV-associated hospitalizations in Alaska Native infants.* Int J Circumpolar Health, 1998. **57 Suppl 1**: p. 255-9.

128. Sonoda, S., et al., *Acute lower respiratory infections in hospitalized children over a 6 year period in Tokyo.* Pediatr Int, 1999. **41**(5): p. 519-24.

129. Souza, L.S., et al., *Viral respiratory infections in young children attending day care in urban Northeast Brazil.* Pediatr Pulmonol, 2003. **35**(3): p. 184-91.

130. Stensballe, L.G., et al., *Acute lower respiratory tract infections and respiratory syncytial virus in infants in Guinea-Bissau: a beneficial effect of BCG vaccination for girls community based case-control study.* Vaccine, 2005. **23**(10): p. 1251-7.

131. Straliotto, S.M., et al., *Viral etiology of acute respiratory infections among children in Porto Alegre, RS, Brazil.* Rev Soc Bras Med Trop, 2002. **35**(4): p. 283-91.

132. Terletskaia-Ladwig, E., et al., *Defining the timing of respiratory syncytial virus (RSV) outbreaks: an epidemiological study.* BMC Infect Dis, 2005. **5**(1): p. 20.

133. Thawatsupha, P., et al., *Isolation and identification of influenza virus strains circulating in Thailand in 2001.* Southeast Asian J Trop Med Public Health, 2003. **34**(1): p. 94-7.

134. Thomas, E., et al., *Respiratory syncytial virus subgroup B dominance during one winter season between 1987 and 1992 in Vancouver, Canada.* J Clin Microbiol, 1994. **32**(1): p. 238-42.

135. Torzillo, P., et al., *Etiology of acute lower respiratory tract infection in Central Australian Aboriginal children.* Pediatr Infect Dis J, 1999. **18**(8): p. 714-21.

136. Tsai, H.P., et al., *Respiratory viral infections among pediatric inpatients and outpatients in Taiwan from 1997 to 1999.* J Clin Microbiol, 2001. **39**(1): p. 111-8.

137. Tseng, R.K., H.Y. Chen, and C.B. Hong, *Influenza virus infections in Taiwan from 1979 to 1995.* Jpn J Med Sci Biol, 1996. **49**(3): p. 77-93.

138. Tsolia, M.N., et al., *Epidemiology of respiratory syncytial virus bronchiolitis in hospitalized infants in Greece.* Eur J Epidemiol, 2003. **18**(1): p. 55-61.

139. Uduman, S.A., et al., *Respiratory syncytial virus infection among hospitalized young children with acute lower respiratory illnesses in Al Ain, UAE.* J Commun Dis, 1996. **28**(4): p. 245-52.

140. Vardas, E., D. Blaauw, and J. McAnerney, *The epidemiology of respiratory syncytial virus (RSV) infections in South African children.* S Afr Med J, 1999. **89**(10): p. 1079-84.

141. Vicente, D., et al., *Hospitalization for respiratory syncytial virus in the paediatric population in Spain.* Epidemiol Infect, 2003. **131**(2): p. 867-72.

142. Vidal, L.R., et al., *The epidemiology and antigenic characterization of influenza viruses isolated in Curitiba, South Brazil.* Mem Inst Oswaldo Cruz, 2008. **103**(2): p. 180-5.

143. Vieira, S.E., et al., *Clinical patterns and seasonal trends in respiratory syncytial virus hospitalizations in Sao Paulo, Brazil.* Rev Inst Med Trop Sao Paulo, 2001. **43**(3): p. 125-31.

144. Videla, C., et al., *Acute lower respiratory infections due to respiratory syncytial virus and adenovirus among hospitalized children from Argentina.* Clin Diagn Virol, 1998. **10**(1): p. 17-23.

145. von Linstow, M.L., et al., *Human metapneumovirus and respiratory syncytial virus in hospitalized danish children with acute respiratory tract infection.* Scand J Infect Dis, 2004. **36**(8): p. 578-84.

146. Wahab, A.A., S.T. Dawod, and H.M. Raman, *Clinical characteristics of respiratory syncytial virus infection in hospitalized healthy infants and young children in Qatar.* J Trop Pediatr, 2001. **47**(6): p. 363-6.

147. Waicharoen, S., et al., *Influenza viruses circulating in Thailand in 2004 and 2005.* Jpn J Infect Dis, 2008. **61**(4): p. 321-3.

148. Waris, M., *Pattern of respiratory syncytial virus epidemics in Finland: two-year cycles with alternating prevalence of groups A and B.* J Infect Dis, 1991. **163**(3): p. 464-9.

149. Weber, M.W., et al., *An epidemiological study of RSV infection in the Gambia.* Bull World Health Organ, 2002. **80**(7): p. 562-8.

150. Weber, M.W., E.K. Mulholland, and B.M. Greenwood, *Respiratory syncytial virus infection in tropical and developing countries.* Trop Med Int Health, 1998. **3**(4): p. 268-80.

151. Weigl, J.A., W. Puppe, and H.J. Schmitt, *The incidence of influenza-associated hospitalizations in children in Germany.* Epidemiol Infect, 2002. **129**(3): p. 525-33.

152. Winter, G.F., et al., *Respiratory viruses in a hospitalized paediatric population in Edinburgh 1985-1994.* J Infect, 1996. **33**(3): p. 207-11.

153. Wong, C.M., et al., *Influenza-associated hospitalization in a subtropical city.* PLoS Med, 2006. **3**(4): p. e121.

154. Yang L, Wong CM, Lau EH, Chan KP, Ou CQ, Peiris JS. [*Synchrony of clinical and laboratory surveillance for influenza in Hong Kong*](http://www.ncbi.nlm.nih.gov/pubmed/18167558). PLoS One. 2008 Jan 2;3(1):e1399

155. Yeolekar, L.R., et al., *Respiratory viruses in acute respiratory tract infections in Western India.* Indian J Pediatr, 2008. **75**(4): p. 341-5.

156. Yun, B.Y., et al., *Viral etiology and epidemiology of acute lower respiratory tract infections in Korean children.* Pediatr Infect Dis J, 1995. **14**(12): p. 1054-9.

157. Zamberi, S., I. Zulkifli, and I. Ilina, *Respiratory viruses detected in hospitalised paediatric patients with respiratory infections.* Med J Malaysia, 2003. **58**(5): p. 681-7.
